# Optimized between-group classification: a new jackknife-based gene selection procedure for genome-wide expression data

**DOI:** 10.1186/1471-2105-6-239

**Published:** 2005-09-28

**Authors:** Florent Baty, Michel P Bihl, Guy Perrière, Aedín C Culhane, Martin H Brutsche

**Affiliations:** 1 Pulmonary Gene Research, University Hospital Basel, CH-4031 Basel, Switzerland; 2 Laboratoire de Biométrie et de Biologie Évolutive, UMR CNRS 5558, Université Claude Bernard Lyon 1, 43 blvd du 11 Novembre, 1918, 69622 Villeurbanne Cedex, France; 3 Bioinformatics Conway Institute, University College Dublin, Ireland

## Abstract

**Background:**

A recent publication described a supervised classification method for microarray data: Between Group Analysis (BGA). This method which is based on performing multivariate ordination of groups proved to be very efficient for both classification of samples into pre-defined groups and disease class prediction of new unknown samples. Classification and prediction with BGA are classically performed using the whole set of genes and no variable selection is required. We hypothesize that an optimized selection of highly discriminating genes might improve the prediction power of BGA.

**Results:**

We propose an optimized between-group classification (OBC) which uses a jackknife-based gene selection procedure. OBC emphasizes classification accuracy rather than feature selection. OBC is a backward optimization procedure that maximizes the percentage of between group inertia by removing the least influential genes one by one from the analysis. This selects a subset of highly discriminative genes which optimize disease class prediction. We apply OBC to four datasets and compared it to other classification methods.

**Conclusion:**

OBC considerably improved the classification and predictive accuracy of BGA, when assessed using independent data sets and leave-one-out cross-validation.

**Availability:**

The R code is freely available [see Additional file 1] as well as supplementary information [see Additional file 2].

## Background

Gene expression microarrays enable the simultaneous measurement of the expression levels of thousands of genes. Supervised classification of gene expression data aims to identify combinations of genes which give the best discrimination of groups of samples specified in advance. For such methods, which are classically used in disease class prediction, the identification of a subset of discriminating genes can be critical [1,2]. Indeed, a large proportion of genes are generally non-informative in terms of disease class prediction. A gain in classification and prediction performance can be expected when predictors are built upon a subset of highly discriminating genes [3,4].

Several algorithms capable of selecting a subset of predictive genes were recently proposed [5]. These methods include a genetic algorithm [6], maximum difference subset algorithm (MDSS) [7], support vector machines [8,9], a shrunken centroids technique [2,10] and several which use of discriminant functions [11].

However, two issues remain: 1) different subsets of genes may provide comparable optimal discriminations [1]; 2) it is generally difficult to determine the optimal number of genes for discrimination [12,13]. This number may vary according to the number of individuals in the training set, the number of groups to discriminate and the method used for classification and prediction. Dolédec and Chessel [14] developed a supervised classification approach, Between Group Analysis (BGA), which was recently applied to microarray data [15]. The authors specified several key features of BGA that make it a method of choice for sample classification and class prediction. In BGA, all genes participate in the discrimination. Consequently, no gene selection step is required. On the other hand, BGA calculates group means and is therefore sensitive to outliers. Our objective was to improve the robustness of BGA by optimizing the number of discriminating genes supporting the analysis.

In this study, we propose a new jackknife-based algorithm – optimized between-group classification (OBC) – that produces a selection of the most robust discriminating genes in order to improve the accuracy of disease class prediction. The criterion optimized in OBC is the percentage of between group inertia (% BG inertia). OBC is applied to BGA but it could also be associated with other supervised methods. We tested the efficiency of OBC on four datasets using independent test sets and leave-one-out cross-validation (LOOCV). We compared our approach to different classification methods.

## Results

### Outline of the OBC algorithm

**Figure 1:**
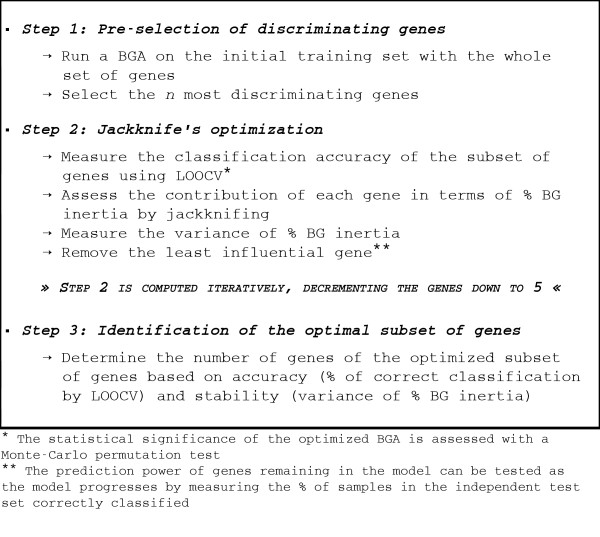
**Overall description of OBC**. Three steps are required to perform OBC optimization. In the first pre-selection step, *n *most discriminating genes are selected by performing a BGA on the training set with the whole set of genes. In the second step, a jackknife optimization is performed on the initial subset of genes and the least influential genes in terms of % BG inertia are removed successively. This second step is iteratively computed, decrementing the genes down to 5. Finally in the third step, the optimal subset of genes is identified (subset with the best classification accuracy and the best stability).

OBC can be described in three steps (Figure 1). These steps are detailed below. Each dataset used in this study was systematically split into a training set and a test set. OBC was applied exclusively to the training set.

### Pre-selection of discriminating genes – step 1

In the first step, pre-selection of a few hundred most discriminating genes is made. This is to reduce the number of calculations and computational resources in step 2 (below). This initial set of discriminating genes is obtained from a BGA of the whole training set (including all genes). Genes with the highest scores on BGA discriminating axes, those located at extremities of BGA axes, are collected. For datasets where samples are grouped into 2 categories (binary categorization), we selected an equal amount of genes at each end of the single discriminating BGA axis. For datasets with more than two categories, we chose genes projected at the periphery of each pair of discriminating axes using a "peeling" function (successive 2D convex hulls).

### Jackknife optimization – step 2

This second step of the algorithm is cpu and time consuming. Due to computational limitations, the number of pre-selected genes should be in the order of a few hundreds (the optimization of 150 genes and 24 samples required 1 h 50 min on a Pentium 4 2.66 GHz computer). Strategies to reduce calculation time are discussed below.

#### Classification accuracy by LOOCV

The performance of the subsets of predictive genes was assessed using LOOCV. To perform LOOCV, a sample is removed from the dataset and a BGA is performed on the remaining samples. The excluded sample is projected on to the BGA and classified. This is iteratively performed until all samples have been subjected to cross-validation. The percentage of samples correctly classified by cross-validation is calculated. This parameter measures the prediction accuracy of the subset of genes.

#### Optimization criterion

The objective of OBC is to improve the discrimination efficiency of BGA, by excluding genes which contribute least to the % BG inertia of samples. OBC uses a jackknife iteration to maximize the between group inertia while minimizing the within group inertia. The inertia decomposition can be described as follow.

Let us suppose *N *the number of samples (*x*_*i *_is the *i*^th ^sample and *w*_*i *_its weight), dist(*x*_*i*_, *x*_*j*_) the squared Euclidean distance between two samples *x*_*i *_and *x*_*j*_, *K *the number of groups (*G*_*k *_is the *k*^th ^group) and *N*_*k *_the number of individuals in the *k*^th ^group. Potentially distances other than Euclidean could be used. In a preliminary analysis we found that the Euclidean distance performs similarly to Manhattan distance. Therefore, given its relative ease of implementation, we use Euclidean distance throughout our analyses. By using a weighted pair-group average calculation, the total inertia can be decomposed into within group inertia (Eq. 1) and between group inertia (Eq. 2). The algorithm aims to maximize the percentage of between group inertia, i.e. the ratio of the between group inertia to the total inertia (Eq. 3).





% BG inertia = BG inertia/(WG inertia + BG inertia)     (3)

#### Measurement of the contribution of each individual gene using jackknifing

We assessed sequentially the influence of each gene in the remaining gene subset using a jackknife procedure. In jackknife analysis, we remove a gene, perform a BGA on the dataset and calculate the % BG inertia. If we remove a gene which positively contributes to the between group discrimination, the % BG inertia decreases and vice versa. By comparing the % BG inertia before and after removing a given gene, one can assess the influence of this gene. In addition, we assess the stability of the % BG inertia during jackknife (described later).

Jackknife approaches have been previously used in the context of gene selection [7,16]. As an example, Lyons-Weiler *et al. *(2003) [7] used jackknifing to reduce the false positive rate of a gene set. In the present study, we used jackknifing to progressively eliminate the least discriminative genes from a subset of genes.

#### Backward optimization

At each step of the algorithm, the gene that contributes least to the % BG inertia is removed from the dataset. Another jackknife procedure is then performed with the remaining genes. This backward optimization algorithm reduces the number of genes from a large subset (typically a few hundreds of genes) to a minimal subset (fixed to minimum of 5).

#### Stability and robustness of the optimization – variance of % BG inertia and Monte-Carlo permutation test

The variance of % BG inertia was used as a measure of the stability of the optimization. By jackknifing a subset of *n *genes, we obtain *n *values of % BG inertia. The range of variation of these values is the variance of % BG inertia. During backward optimization the number of genes included in the classifier gets smaller, and the effect of the jackknife perturbation measured by the variance of % BG inertia tends to increase. If this variance is high, the robustness and the stability of the prediction model is low. Consequently, low variance of % BG inertia is preferable.

Throughout the optimization, the statistical significance of BGA is evaluated with a Monte-Carlo permutation test.

### Identification of the optimal subset of genes for disease class prediction – step 3

The optimal subset of genes are identified with the aid of the summary diagram which summarizes the results of the algorithm. The optimal subset of genes should have both high LOOCV prediction accuracy and stability (i.e. minimal variance of % BG inertia). If optimization of these two parameters resulted in a range of near optimal solutions, we chose subsets with fewer genes and higher % BG inertia. Importantly, although we calculate prediction accuracy of the independent test set, these results were never taken into account in OBC, as this would result in over-training.

### Application of OBC to sarcoidosis data

#### Between group analysis

**Figure 2:**
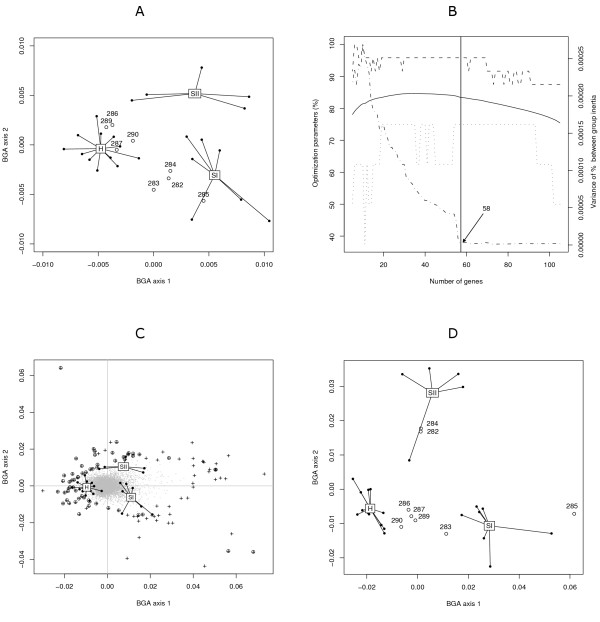
**Optimized between-group classification applied to sarcoidosis data**. In panel A, 24 individuals (solid circles) in the training set (H: healthy controls, SI: sarcoidosis stage I, SII: sarcoidosis stage II/III) and 8 individuals (empty circles) in the test set (283, 286, 287, 289 and 290 as H; 282, 284 and 285 as SII) are classified by a standard BGA using the whole set of genes. Panel B shows the different parameters of OBC as a function of the number of genes used in the analysis: the percentage of between group inertia (solid line), the percentage of good cross-validation (dashed line) and the variance of between group inertia (dot-dashed line). For indication, the percentage of test samples correctly predicted is represented by a dotted line. This parameter was not used in optimization of the training model. The vertical line shows the optimal number of genes. In panel C, the 105 most discriminating genes (initial subset) are located at the periphery of the biplot (black crosses) and the 58 optimal genes are highlighted (circled crosses). In panel D, 8 test-samples are classified using a BGA based on the 58 optimal genes.

Standard BGA was applied to the whole sarcoidosis training data set. The biplot representation shows that BGA separated the three phenotypes with no overlap (Figure 2, panel A). The first axis separated the healthy controls from the sarcoidosis patients. The second axis separates the two stages of sarcoidosis. The efficiency of classification of new samples was measured using LOOCV. Seventy-five percent of the 24 samples were classified correctly. However, we observed discrepancies in classification accuracy between the three phenotypes. All healthy controls, 6 out of 7 stage I, but none of the stage II/III sarcoidosis patients were correctly re-classified. When we tried to predict the classification of a blind test of 8 follow-up patients, using this BGA of the whole set of genes, only 50% of these test samples were correctly classified. Four out of five patients, which recovered 6 months after they were diagnosed with a stage I sarcoidosis, were classified in the healthy group. All of the patients still suffering from active sarcoidosis stage II/III (*n *= 3) were incorrectly classified.

#### Optimized between group classification

We selected the 105 most discriminating genes in this initial BGA, using the above mentioned peeling procedure (Figure 2, panel C). OBC was applied on this subset of genes. The least influential genes in terms of % BG inertia were removed one by one.

Figure 2 (panel B) shows the evolution of classification parameters; % BG inertia, % correct classification in LOOCV, and variance of % BG inertia. During the optimization process, the % BG inertia increased when the number of genes decreased until it reached an optimum, then it decreased when the number of genes fell below this optimum threshold. The percentage of correct classification in LOOCV was stable in a range of 20–70 genes. When the number of genes further decreased, it started to oscillate. The variance of % BG inertia was very low for subsets of more than 58 genes. This parameter increased considerably for subsets fewer than 57 genes. Finally, the dotted line represents the evolution of percentage of test sets correctly classified (this parameter was not considered during optimization).

The subset of genes with the best cross-validation efficiency and least variable % BG inertia was judged to be the optimal subset. Therefore, this was a subset of 58 genes (Figure 2, panel C). The accuracy of LOOCV obtained using this optimized subset of genes was clearly improved since 96% of samples were correctly classified (100, 80 and 100% respectively in sarcoidosis stage I, stage II/III and healthy controls). Figure 2 (panel D) shows the projection of 8 follow-up samples predicted by this subset of classifiers. These predictions were also improved since 2/3 of sarcoidosis stage II/III were correctly associated to their group, whereas 4/5 of patients in remission from a stage I sarcoidosis were classified as healthy. Patient 283, who was mis-classified, clinically recovered from a sarcoidosis stage I. It is possible that signals of gene activity specific to stage I sarcoidosis be still detectable in this patient.

### Application of OBC to tumour data

#### Between group analysis

**Figure 3:**
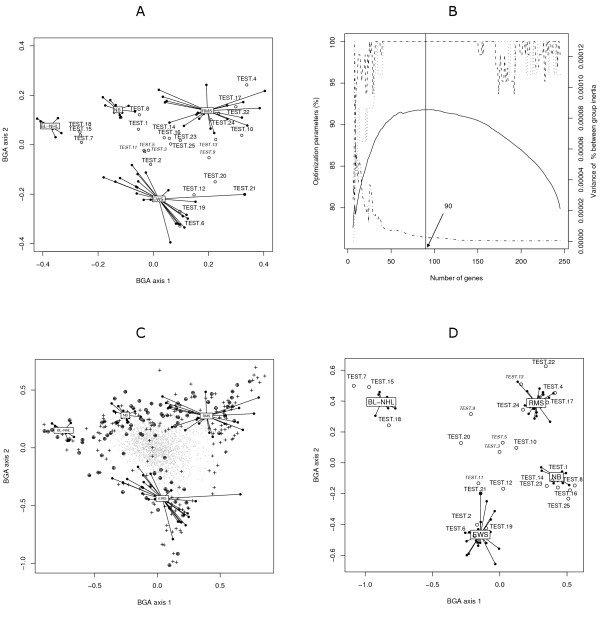
**Optimized between-group classification applied to tumour data**. In panel A, 63 samples (solid circles) of the training set (BL: Burkitt's lymphoma, EWS: Ewing's sarcoma, NB: neuroblastoma, RMS: rhabdomyo sarcoma) and 25 samples (empty circles) of the test set (7, 15 and 18 as BL-NHL; 2, 6, 12, 19, 20 and 21 as EWS; 1, 8, 14, 16, 23 and 25 as NB; 4, 10, 17, 22 and 24 as RMS; 3, 5, 9, 11 and 13 as control samples that do not belong to one of the 4 groups) are classified by the standard BGA based on the whole set of genes. Panel B shows the different parameters of OBC as a function of the number of genes used in the analysis: the percentage of between group inertia (solid line), the percentage of good cross-validation (dashed line) and the variance of between group inertia (dot-dashed line). For indication, the percentage of test samples correctly predicted is represented by a dotted line. This parameter was not used in optimization of the training model. The vertical line shows the optimal number of genes. In panel C, the 245 most discriminating genes are represented with small crosses and the 90 optimal genes are highlighted (circled crosses). In panel D, the 25 test-samples are classified using a BGA based on the 90 optimal genes.

BGA was applied to the whole tumour training set [20]. BGA clearly separated the 4 different types of tumours with no overlap (Figure 3, panel A). Based upon the complete set of 2308 genes, the LOOCV showed that 93% of the 63 samples from the training set were correctly cross-validated and 19/20 of the test sets were correctly predicted. The most discriminating genes associated with the different groups were identified at the periphery of the BGA biplot (Figure 3, panel C).

#### Optimized between-group classification

From the initial BGA, the 245 most discriminating genes were selected. We applied the optimization algorithm to this initial subset. We used the optimization diagram to determine the optimal subset of genes. As shown in diagram Figure 3, panel B, there was a range of near optimal solutions (high % of correct cross-validation and low variance of % BG inertia). We decided to choose an optimal subset of 90 genes for which the accuracy, the stability and the % BG inertia were high.

The results of BGA using the 90 optimal genes are plotted in Figure 3 (panel C). The accuracy of LOOCV, of BGA on the 63 training samples using the 90 optimal genes, increased to 100%. All 20/20 test sets were correctly classified (Figure 3 panel D).

### Stability of OBC and test of significance

The stability of OBC was controlled by monitoring the evolution of variance of % BG inertia. This parameter was of great importance as it monitored whether the classification was overly influenced by a few genes.

The Monte-Carlo permutation test was constantly significant for the different datasets (estimated *p*-value = 0.001). This result suggests that our method is robust.

### Sensitivity and specificity

We built confusion matrices from the results obtained from LOOCV and classification of independent test sets. Then, we calculated the sensitivity and specificity of BGA and OBC for each disease category. Sensitivity measures the proportion of individuals correctly classified for a given disease class (true positives). Specificity measures the proportion of individuals that do not belong to the class and which are not classified in this class (true negatives). The sensitivities and specificities of OBC vs. standard BGA are summarized in Tables 1 and 2.

**Table 1:** Sensitivity and specificity of OBC compared with standard BGA in sarcoidosis dataset.

		LOOCV			Test set		
		H	SI	SII	H	SI	SII

OBC	Sensitivity	1	1	0.8	0.8	-	0.67
	Specificity	0.92	1	1	1	0.5	1

BGA	Sensitivity	1	0.86	0	0.8	-	0
	Specificity	0.83	0.82	0.95	1	0.75	1

**Table 2:** Sensitivity and specificity of OBC compared with standard BGA in tumour dataset.

		LOOCV				Test set			
		EWS	NB	RMS	BL	EWS	NB	RMS	BL

OBC	Sensitivity	1	1	1	1	1	1	1	1
	Specificity	1	1	1	1	1	1	1	1

BGA	Sensitivity	0.87	1	0.9	1	1	0.83	1	1
	Specificity	0.975	0.98	0.93	1	1	1	0.93	1

**Figure 4:**
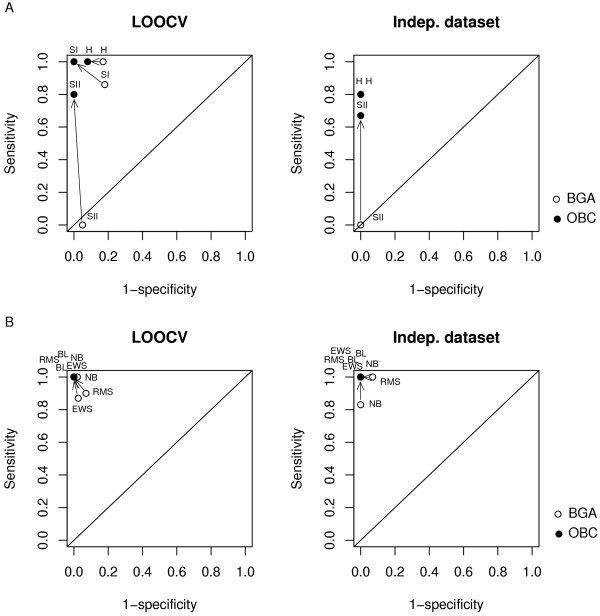
**Analysis of sensitivity and specificity**. The sensitivity and specificity of OBC (solid circles) were compared to standard BGA (empty circles). The prediction accuracy of OBC when applied to the sarcoidosis dataset was assessed using (A) LOOCV (left panel) and classification of the independent dataset (right panel). OBC was also applied to the tumour dataset and tested using (B) LOOCV (left panel) and classification of the independent dataset (right panel). Arrows show the improvement of sensitivity and specificity obtained with OBC compared to the standard BGA.

Figure 4 shows the sensitivity as a function of 1 – specificity of BGA with and without optimization (black dots and white dots, respectively), when applied to the sarcoidosis and tumour datasets (panels A and B, respectively). The results of LOOCV and classification of independent test sets are shown in the left and right plots respectively.

We observed an improvement in prediction sensitivity and specificity of both the sarcoidosis (Figure 4A) and tumour datasets (Figure 4B) when OBC was applied in LOOCV and independent test sample cross validation.

### Comparison with other algorithms

We compared OBC with three other recently described gene selection methods: the GA/KNN algorithm [6], maximal margin linear programming (MAMA) [17] and nearest shrunken centroid [10]. Results (Table 3) show that OBC outperforms these approaches in terms of accuracy of LOOCV and classification of independent test sets. Comparisons between BGA and other supervised classification methods [15] report that BGA outperforms or performs with similar effectiveness.

**Table 3:** Comparison of the accuracy of OBC with different classification methods.

Dataset	Method	% correct LOOCV	% correct prediction of independent test samples
Sarcoidosis	OBC	96%	75%
	BGA	75	50
	GA/KNN	92	62.5
	MAMA	67	62.5
	Shrunken centroids	79^1^	62.5^1^
Tumour	OBC	100	100
	BGA	92.6	95
	GA/KNN	100	95
	MAMA	98	76
	Shrunken centroids	100^2^	90^2^

^1^Threshold of 2.801

^2^Threshold of 2.459

### OBC applied to datasets with binary categorization

#### Colon cancer dataset

We assessed the prediction accuracy of OBC when applied to the colon cancer data set, which contains two categories of tumor samples. We applied OBC optimization to the 100 most discriminating genes. Results of LOOCV, show an increase of accuracy from 85% for standard BGA to 94% for OBC (based on 20 optimized genes). We investigated the sensitivity and specificity of OBC classification prediction when applied to independent test data. We built 26 pairs of training sets/test sets by randomly splitting the complete data set of 62 samples into training sets of 40 samples and test sets of 22 samples. OBC produced an improvement in both the sensitivity (83% to 87%) and specificity (87% to 91%) of prediction.

#### Leukemia dataset

We compared the prediction accuracy of BGA and OBC using LOOCV of the whole dataset. The percentage of samples correctly predicted in LOOCV was 90% for BGA and 99% for OBC (based on 40 optimized genes). Similarly to the colon cancer data analysis, we built 24 pairs of training sets/test sets by randomly splitting the whole dataset into 50 training and 22 test samples. Application of OBC to the leukemia dataset improved the sensitivity and specificity of test set classification. When OBC was applied, the sensitivity of classification was improved for both ALL (97% to 99%) and AML (91% to 92%). The specificity of prediction of both ALL and AML samples was also improved (respectively, 91% to 92% and 97% to 99%).

## Discussion

Selection of genes that optimize disease class prediction is a significant and difficult challenge in microarray data analysis. Most discriminative functions require more cases than variables which is not realistic in the context of microarray experiments. A further challenge is the considerable amount of noise in microarray data. BGA can be applied to complete datasets without prior gene selection and performs comparably or outperforms several other approaches [15]. We showed that an optimized gene selection considerably improves the predictive power of BGA. Our jackknife-based algorithm tests the robustness of BGA discriminating genes and progressively excludes weaker discriminators. As a consequence, it optimizes the performance of BGA and reduces the number of discriminating genes.

**Figure 5:**
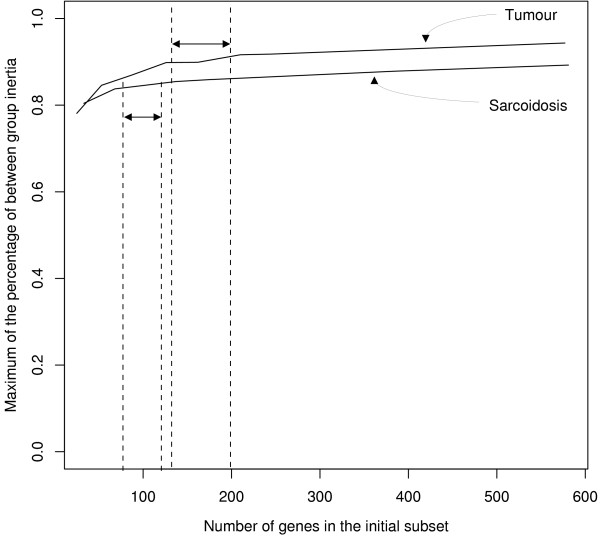
**Number of genes included in the initial subset**. This plot shows the maximum % BG inertia reached by the optimization procedure as a function of the number of genes present in the initial subset of genes (top curve: tumour data; bottom curve: sarcoidosis data). The dashed lines delimit the optimal size of the initial subset of genes for both datasets (above which the gain in % BG inertia is lower).

The OBC algorithm presented here might be time consuming depending on the size of the initial subset of genes. Increasing the number of genes in the initial dataset ensures that more potentially discriminative genes are present in the analysis. However, the time required for the optimization process increases significantly. We assessed the percentage of gain in % BG inertia obtained by increasing the number of genes in the initial subset. This number depends on the dataset and in particular the number of groups to discriminate. The optimal number of genes of OBC starting genes is around 100 for the sarcoidosis data and 150–200 in the tumour data (Figure 5).

In OBC, the choice of the initial subset of genes from which the algorithm starts remains critical and alternative procedures might be used. For example, the genetic algorithm proposed by Li *et al. *(2001) [6] could be associated to OBC and might provide some improvements in performance.

Different options could be considered to speed up the algorithm. We considered removing more than one least influential gene at a time in the jackknife optimization. The execution time would decrease proportionally to the number of genes removed at each step. For example, if we removed 10 % least influential genes from the subset of genes at each step, we could greatly increase the speed of execution of the algorithm. With this, it would be possible to include a few thousands of genes in the initial subset of genes. The decision on how many genes to remove per jackknife cycle is a trade off between testing more combinations of genes (and therefore testing more efficiently gene-gene interactions) and including more genes in the analysis. On the other hand, the numerous tasks performed during the optimization could be split into several jobs, which could be potentially computed in parallel by a cluster of processors/computers. Finally another solution would be to rewrite the computationally demanding parts of the algorithm in a more efficient computer language like C.

We decided to choose a backward optimization procedure as this seemed to be more adapted for taking possible gene-gene interactions into account. The prediction power of a single gene might be negligible in itself while it might be preponderant when associated with one or a few other genes. Removing a gene that jointly participates with other genes to the group discrimination will have an impact, which is measurable by a backward approach, whereas no evidence might be found by using a forward optimization.

Our results show that an improvement in discriminative and predictive power of BGA can be achieved by reducing the number of predictors in the analysis to a small subset of highly discriminative genes. These genes contribute to improve the % BG inertia. In this study, two criteria were used to define the optimal subset of genes: a positive criterion, the percentage of correct classification by LOOCV and a negative criterion the variance of % BG inertia. When searching for the subset of genes where both criteria were optimized, we generally found a range of near optimal solutions. In the sarcoidosis dataset, the size of the optimal subset of genes was around 60, whereas in the tumour dataset, subsets including around 90 genes were found to be optimal. By using a method that associates a genetic algorithm with the k-nearest neighbors technique (GA/KNN) on a lymphoma dataset, Li *et al. *(2001) [6] concluded that using only a few discriminating genes may not be reliable, whereas using too many genes will add noise to the classification. They suggested 50–200 genes would give an optimal result which is in agreement with our study.

## Conclusion

We propose OBC, a novel jackknife-based backward optimization algorithm, which improves both the classification and predictive power of BGA. Our algorithm tended to outperform alternative classification techniques. In the future, OBC could be used as a decision making-tool for disease class prediction based on gene expression data in various clinical situation. Future developments will include the application of the algorithm to different supervised methods.

## Methods

### Data sets

#### Sarcoidosis data

The gene expression study was carried out on 12 healthy controls (H), 7 sarcoidosis stage I patients (SI) and 5 sarcoidosis stage II/III patients (SII). This dataset was published previously and details can be found in [18]. These 24 samples correspond to the sarcoidosis training set. In addition, 6 months later, 8 follow-up chips were done for some of the sarcoidosis patients. Among these patients, 3 still had active sarcoidosis stage II/III and 5 were recovered from sarcoidosis stage I. These 8 supplementary samples correspond to the sarcoidosis test set. The expression level of 12626 probe sets was measured with Affymetrix' GeneChip^® ^(HG-U95Av2). The complete dataset and the raw files have been deposited in NCBIs Gene Expression Omnibus (GEO) [19], and are accessible through GEO Series accession number GSE1907.

#### Tumour data

This dataset was published by Khan et al. (2001) [20]. The authors measured the expression of 6567 genes in four types of small round blue cell tumours (NB: neuroblastoma; RMS: rhabdomyo sarcoma; BL: Burkitt's lymphoma; EWS: Ewing's sarcoma). A filtered dataset containing the expression level of 2308 genes is publicly accessible [21]. The whole dataset contained 88 samples split into a training set (63 samples) and a test set (25 samples).

#### Colon cancer data

This colon cancer dataset was studied by Alon et al. (1999) [22]. It contained 62 samples obtained from 40 tumor samples and 22 control samples. Gene expression profiles were analyzed using Affymetrix' microarrays containing more than 6500 genes. This dataset was randomly split into training sets and test sets (40 and 22 samples, respectively). This dataset is available as a Bioconductor data package [23].

#### Leukemia data

The leukemia dataset [24] contained 72 samples from patients having two types of acute leukemia. Among the 72 patients, 47 had acute lymphoblastic leukemia (ALL) and 25 had acute myeloid leukemia (AML). Samples were obtained from bone marrow or peripheral blood. Gene expression profiling was analyzed with Affymetrix' microarrays containing 7159 probe sets. This dataset was randomly split into training sets and test sets (50 and 22 samples, respectively). The dataset is available as a Bioconductor data package [25].

### Software and statistical analysis

The OBC algorithm was written in R (version 1.9.1), an open-source statistical software [26]. The algorithm is freely available [see Additional file 1] and further information can be find as well [see Additional file 2]. Some specific R packages were used in this study: the Bioconductor packages for microarray analysis [27]; ADE4 [28] and MADE4 [29] for multivariate analysis. The sarcoidosis dataset was normalized using the *vsn *algorithm [30].

### Between group analysis

BGA is a particular extension of conventional ordination methods such as principal component analysis (PCA) or correspondence analysis (COA) where groups of samples are specified in advance [14]. The association of COA with BGA is particularly powerful, as COA has been shown to have several advantages over PCA in analysis of gene expression data [31,32]. In order to simplify the notations in the paper, the acronym BGA refers to the between-group correspondence analysis.

The between group analysis of the statistical triplet (*X*, *Q*, *D*) – where *X *is a data table of *n *rows (samples) and *p *columns (variables), Q is a *p *× *p *diagonal matrix containing the variable weights and D is a *n *× *n *diagonal matrix containing the sample weights – given the class indicator *f*, is the analysis of the triplet (*G*, *Q*, *D*_*w*_) where *G *is the table of the means of *X *per group and *D*_*w *_is the diagonal matrix of group weights [28]. Let us consider *K *the number of specified groups, a typical BGA yields *K *- 1 discriminating axes that ordinate the groups of sample by maximizing the between group variance (see [15] for mathematical details). Linear discriminant analysis is a related method which aims to maximize the percentage of variance explained by the grouping but which has different constraints and which cannot be applied to tables where the number of variables exceeds the number of samples [33].

Genes and samples ordinated by BGA can be projected on discriminating axes and visualized simultaneously on a biplot. The most discriminating genes are projected at the extremity of each axis whereas less informative genes are projected near the origin of each axis.

## Authors' contributions

FB developed the algorithm, performed the analysis and wrote the paper in the team led by MHB. MHB supervised the study and provided substantial methodological input and was involved in drafting of the manuscript. MPB gave technical advice and biological input AC and GP provided important support regarding the refinements of the algorithm and BGA. All authors read and approved the manuscript.

## Supplementary Material

Additional File 1 R code of the OBC algorithm. Click here for file

Additional File 2 Further description of the sarcoidosis and tumour data. This files gives details about the optimal subset of genes obtained after OBC. Click here for file
