# Supplementary material for: Optimized between-group classification: a new jackknife-based gene selection procedure for genome-wide expression data
Source: BMC Bioinformatics. 2005 Sep 28;6:239. doi: 10.1186/1471-2105-6-239 (PMC1261161; doi:10.1186/1471-2105-6-239)
Supplement: Additional File 2 — Further description of the sarcoidosis and tumour data. This files gives details about the optimal subset of genes obtained after OBC. [file 1471-2105-6-239-S2.pdf]

Optimized between-group classification: a new jackknife-based gene selection procedure for genome-wide expression data

Supplementary information

Florent Baty*[1] florent.baty@unibas.ch
Michel P Bihl[1] michel.bihl@unibas.ch
Guy Perrière[2] perriere@biomserv.univ-lyon1.fr
Aedín C Culhane[3] Aedin.Culhane@ucd.ie
Martin H Brutsche[1] mbrutsche@uhbs.ch

[1]Pulmonary Gene Research, University Hospital Basel, CH-4031 Basel, Switzerland
[2]Laboratoire de Biométrie et de Biologie Évolutive, UMR CNRS 5558, Université Claude Bernard Lyon 1, 43 blvd du 11 Novembre 1918, 69622 Villeurbanne Cedex, France
[3]Bioinformatics Conway Institute, University College Dublin, Ireland

Algorithm

R code

The R code is freely available. For further information regarding the R code please contact Florent Baty: florent.baty@unibas.ch

Sarcoidosis dataset

Experimental design

Type of experiment	Healthy controls vs. Sarcoidosis stage I patients vs. Sarcoidosis stage II/III patients
Experimental factors	Same as above in peripheral blood cells
Number of hybridizations performed	32
Type of reference used for the hybridizations	N/A
Hybridization design	See table below
Quality control	O/R
URL	http://www.ncbi.nlm.nih.gov/projects/geo/query/acc.cgi?acc=GSE1907&v

Sample used, extract preparation and labeling

Origin of Biological Sample	All tissues are human
Manipulation of Biological Samples	PAXgene System (PreAnalytiX, Switzerland)
Hybridization Preparation	IVT labeling kit (Affymetrix, Inc., Santa Clara, CA)
Labeling	Standard Affymetrix Protocol (Affymetrix, Inc., Santa Clara, CA)
External controls (spikes)	Standard Affymetrix Protocol (Affymetrix, Inc., Santa Clara, CA)

Hybridization procedures and parameters

Hybridization, Blocking and Washing	Standard Affymetrix Protocol (Affymetrix, Inc., Santa Clara, CA)

Measurement data and specifications

Image Quantitation	Affymetrix scanner to generate CEL file
Set of Quantitation	Data were normalized using the 'vsn' algorithm (PM-only, Medianpolish summary)

Array design

General array design	Affymetrix HGU-95Av2 GeneChip (Affymetrix, Inc., Santa Clara, CA)

Sample information

Phenotype group	Patient ID	Age	Sex	Treatment	Cancer	Inflammation	Follow-up
Stage I	253	23	m	none	none	Sarcoidosis	no
Stage II/III	254	25	m	none	none	Sarcoidosis	yes
Stage I	255	53	f	none	none	Sarcoidosis	yes
Stage II/III	256	34	f	none	none	Sarcoidosis	no
Stage II/III	257	40	f	none	none	Sarcoidosis	yes
Stage II/III	258	61	f	none	none	Sarcoidosis	yes
Stage I	259	20	f	none	none	Sarcoidosis	yes
Stage I	260	27	m	none	none	Sarcoidosis	yes
Stage I	261	38	m	none	none	Sarcoidosis	yes
Stage I	262	39	m	none	none	Sarcoidosis	yes
Stage II/III	263	37	f	none	none	Sarcoidosis	no
Healthy	265	—	—	none	none	Healthy	no
Healthy	266	—	—	none	none	Healthy	no
Healthy	267	—	—	none	none	Healthy	no
Healthy	268	—	—	none	none	Healthy	no
Healthy	269	—	—	none	none	Healthy	no
Healthy	270	—	—	none	none	Healthy	no
Healthy	271	—	—	none	none	Healthy	no
Healthy	272	—	—	none	none	Healthy	no
Healthy	273	—	—	none	none	Healthy	no
Healthy	274	—	—	none	none	Healthy	no
Healthy	275	—	—	none	none	Healthy	no
Healthy	276	—	—	none	none	Healthy	no

Genes selected by optimization

Affy ID	Gene symbol	Gene description
36108_at	HLA-DQB1	major histocompatibility complex, class II, DQ beta 1
37285_at	ALAS2	aminolevulinate, delta-, synthase 2 (sideroblastic/hypochromic anemia)
37864_s_at	IGHG3	immunoglobulin heavy constant gamma 3 (G3m marker)
36280_at	GZMK	granzyme K (serine protease, granzyme 3; tryptase II)
432_s_at	TRA@	T cell receptor alpha locus
39248_at	AQP3	aquaporin 3
38759_at	BTN3A2	butyrophilin, subfamily 3, member A2
37988_at	CD79B	CD79B antigen (immunoglobulin-associated beta)
37984_s_at	ARF6	ADP-ribosylation factor 6
1973_s_at	MYC	v-myc myelocytomatosis viral oncogene homolog (avian)
1097_s_at	CCR7	chemokine (C-C motif) receptor 7
35712_at	LRRN3	leucine rich repeat neuronal 3
36227_at	IL7R	interleukin 7 receptor
40749_at	MS4A1	membrane-spanning 4-domains, subfamily A, member 1
40040_at	MYOM2	myomesin (M-protein) 2, 165kDa
32287_s_at	KLRC3	killer cell lectin-like receptor subfamily C, member 3
38578_at	TNFRSF7	tumor necrosis factor receptor superfamily, member 7
40738_at	CD2	CD2 antigen (p50), sheep red blood cell receptor
35449_at	KLRB1	killer cell lectin-like receptor subfamily B, member 1
34023_at	FCER1A	Fc fragment of IgE, high affinity I, receptor for; alpha polypeptide
619_s_at	MS4A1	membrane-spanning 4-domains, subfamily A, member 1
36878_f_at	HLA-DQB1	major histocompatibility complex, class II, DQ beta 1
41260_at	DDX17	DEAD (Asp-Glu-Ala-Asp) box polypeptide 17
36979_at	SLC2A3	solute carrier family 2 (facilitated glucose transporter), member 3
32035_at	HLA-DRB4	major histocompatibility complex, class II, DR beta 4
40083_at	JARID1D	Jumonji, AT rich interactive domain 1D (RBP2-like)
32598_at	NELL2	NEL-like 2 (chicken)
1106-s_at	TRA@	T cell receptor alpha locus
37209_g_at	PSPHL	phosphoserine phosphatase-like
40511_at	GATA3	GATA binding protein 3
40396_at	P2RX5	purinergic receptor P2X, ligand-gated ion channel, 5
36887_f_at	KIR3DL1	killer cell immunoglobulin-like receptor, three domains, long cytoplasmic tail, 1
36886_f_at	KIR2DL3	killer cell immunoglobulin-like receptor, two domains, long cytoplasmic tail, 3
32066_g_at	CREM	cAMP responsive element modulator
279_at	NR4A1	nuclear receptor subfamily 4, group A, member 1
1061_at	IL10RA	interleukin 10 receptor, alpha
35442_at	KIAA0792	KIAA0792 gene product
32034_at	ZNF217	zinc finger protein 217
37828_at	FLJ11220	hypothetical protein FLJ11220
326_i_at	RPS20	ribosomal protein S20
38242_at	BLNK	B-cell linker
34098_f_at	ITGB1BP1	integrin beta 1 binding protein 1
37399_at	AKR1C3	aldo-keto reductase family 1, member C3 (3-alpha hydroxysteroid dehydrogenase, type II)
37137_at	GZMB	granzyme B (granzyme 2, cytotoxic T-lymphocyte-associated serine esterase 1)
41386_i_at	KIAA0346	KIAA0346 protein
37952_at	ITGB3	integrin, beta 3 (platelet glycoprotein IIIa, antigen CD61)
1148_s_at	NRG1	neuregulin 1
35885_at	USP9Y	ubiquitin specific protease 9, Y-linked (fat facets-like, Drosophila)
39879_s_at	KIAA0563	KIAA0563 gene product
34962_at		
39507_at	OGT	O-linked N-acetylglucosamine (GlcNAc) transferase (UDP-N-acetylglucosamine
2001_g_at	ATM	ataxia telangiectasia mutated (includes complementation groups A, C and D)
36786_at	MKRNP2	makorin, ring finger protein, pseudogene 2
33555_at	ILT7	leukocyte immunoglobulin-like receptor, subfamily A (without TM domain), member 4
38446_at		
39878_at	PCDH9	protocadherin 9
37467_at	IGHD	immunoglobulin heavy constant delta

Tumour dataset

Experimental design

Link:	http://research.nhgri.nih.gov/microarray/Supplement

Genes selected by optimization

Gene symbol
PTPN13
NA
FCGRT
MYC
ELF1
LYN
CDH2
HLA-DMA
GAP43
HCLS1
MME
PBX3
FGFR4
IFI16
PSMB8
CYFIP2
SLC2A1
CNN3
SGCA
NME2
MYC
MT1X
MYO1B
LOXL2
MEST
FABP4
AF1Q
CSRP2
BIN1
NF2
FLJ90440
NA
WAS
GATM
CRMP1
LRBA
NA
IGLL1
MAP1B
CNGB1
RNPC1
PLAU
TXNRD1
IDI1
CD83
EHD1
NA
MYO1B
NEF3
PFKP
PMS2L6
ARPC1B
TCF7L2
NA
PCOLCE
CRABP1
ADA
CDK2AP1
SERPINE1
DPYSL2
ANXA1
ATP1A1
MGST1
RGS16
DNMT2
NRP2
FADS1
PTPN12
IGFBP3
CRI1
TAP1
NFKB1
MEIS1
ATF3
HMGCL
NA
NA
NA
NA
HOXB7
CCNG2
ALDH1B1
C5orf13
MMP2
MGC35097
PAFAH1B3
CITED2
NRGN
CUTL1
DYRK2
